# Mechanical Ventilation Practices in Traumatic Brain Injury: A Systematic Review of Contemporary Evidence

**DOI:** 10.7759/cureus.115663

**Published:** 2026-09-02

**Authors:** Maqdad Taaqi, Shareefah Almutairi, Roza Bahwaini, Waqas Alauddin

**Affiliations:** 1 Physiology, Health Sciences Center - Kuwait University, Kuwait City, KWT; 2 Physical Therapy, Health Sciences Center - Kuwait University, Kuwait City, KWT; 3 Respiratory Therapy, Chest Diseases Hospital, Kuwait City, KWT; 4 Physiology, Venkateswara Institute of Medical Sciences, Amroha, IND

**Keywords:** airway management, intensive care, lung-protective ventilation, mechanical ventilation, neurocritical care, tracheostomy, traumatic brain injury

## Abstract

Traumatic brain injury (TBI) frequently necessitates mechanical ventilation to maintain adequate gas exchange, protect the airway, and support cerebral physiology during the acute phase of critical illness. Although ventilatory management is an integral component of neurocritical care, the optimal approach remains uncertain because interventions aimed at pulmonary protection may influence intracranial dynamics and neurological outcomes. This systematic review aimed to critically evaluate contemporary evidence on mechanical ventilation practices in adults with TBI, with emphasis on ventilatory strategies, lung-protective ventilation, airway management, tracheostomy, weaning approaches, and clinically relevant outcomes. A systematic literature search was conducted in accordance with the Preferred Reporting Items for Systematic Reviews and Meta-Analyses (PRISMA) 2020 guidelines using PubMed/MEDLINE, Scopus, Embase, Web of Science, Cumulative Index to Nursing and Allied Health Literature (CINAHL), EBSCOhost, the Cochrane Central Register of Controlled Trials (CENTRAL), Google Scholar, and selected grey-literature sources for studies published from January 2000 through June 2026. Eligible studies included adult patients with TBI who were receiving mechanical ventilation and reported ventilation-related interventions or outcomes. Methodological quality and risk of bias were assessed using the Newcastle-Ottawa Scale, Joanna Briggs Institute Critical Appraisal Checklists, and the Cochrane Risk of Bias 2 tool, as appropriate for the study design. Owing to substantial clinical and methodological heterogeneity, findings were synthesized narratively. Eleven studies, comprising randomized controlled trials, prospective and retrospective cohort studies, and cross-sectional surveys, met the inclusion criteria. Considerable variation was observed in ventilatory management, particularly regarding tidal volume, positive end-expiratory pressure, carbon dioxide targets, airway management, and tracheostomy timing. The available evidence suggests that individualized ventilatory management may help balance pulmonary protection with preservation of cerebral physiology when guided by appropriate neurological and respiratory monitoring. Early tracheostomy may be associated with shorter durations of mechanical ventilation and intensive care unit stay in selected patients, although consistent benefits in mortality or long-term neurological recovery have not been established. Overall, the included studies demonstrated substantial variability in ventilatory practices and clinical outcomes across different settings and patient populations. Current evidence suggests that ventilation strategies in adults with TBI should be tailored to individual respiratory mechanics and cerebral physiological status rather than applied uniformly. However, substantial heterogeneity, limited TBI-specific comparative evidence, and variation in clinical practice limit the certainty of the available evidence. High-quality multicenter studies are needed to establish evidence-based ventilatory targets and determine their effects on patient-centered neurological and pulmonary outcomes.

## Introduction and background

Traumatic brain injury (TBI) is a major global cause of mortality and long-term neurological disability, placing a substantial burden on healthcare systems, patients, and society [1,2]. An estimated 69 million individuals sustain TBI annually, with a particularly high burden in low- and middle-income countries (LMICs), where road traffic injuries, falls, and interpersonal violence contribute substantially to disease incidence [1,2]. Patients with moderate-to-severe TBI frequently require intensive care because impaired consciousness, loss of protective airway reflexes, and respiratory dysfunction may necessitate advanced airway management and mechanical ventilation. In this setting, prevention of secondary brain injury through careful maintenance of systemic and cerebral physiological parameters remains a central objective of neurocritical care [1,2].

Mechanical ventilation is an important component of supportive management in severe TBI, with implications extending beyond pulmonary gas exchange. Ventilatory settings can influence arterial oxygen and carbon dioxide levels, cerebral blood flow, intracranial pressure (ICP), cerebral perfusion pressure (CPP), and overall cerebral homeostasis [3,4]. Consequently, ventilatory management requires careful consideration of both pulmonary and neurological physiology. Strategies designed to minimize ventilator-induced lung injury may alter intrathoracic pressure and cerebral venous drainage, whereas interventions aimed at controlling intracranial hypertension may increase the risk of pulmonary complications. Balancing pulmonary protection with preservation of cerebral perfusion therefore represents a continuing challenge in the management of patients with severe TBI [3,4].

The management of ventilation in TBI has evolved considerably over time. Hyperventilation was historically used routinely to reduce ICP through hypocapnia-induced cerebral vasoconstriction; however, sustained hypocapnia can decrease cerebral blood flow and potentially aggravate cerebral ischemia [5,6]. Contemporary recommendations therefore generally discourage prolonged prophylactic hyperventilation and emphasize maintaining adequate oxygenation and avoiding clinically significant disturbances in arterial carbon dioxide while addressing intracranial hypertension when clinically indicated [5,6]. Ventilatory targets should consequently be individualized according to the patient's neurological status, cerebral physiology, and respiratory requirements.

Increasing attention has been directed toward the application of lung-protective ventilation in patients with acute brain injury. Lower tidal volumes, individualized positive end-expiratory pressure (PEEP), appropriate oxygenation targets, and carefully selected recruitment strategies may reduce pulmonary complications while maintaining cerebral physiological stability in appropriately monitored patients [7-9]. However, the available evidence is not entirely consistent, and concerns remain regarding the effects of higher PEEP, lower tidal volumes, carbon dioxide alterations, and recruitment maneuvers on ICP and cerebral perfusion in patients with impaired intracranial compliance. Furthermore, substantial variation persists among institutions and healthcare systems in ventilator settings, carbon dioxide targets, airway management, tracheostomy timing, and weaning practices [7-9]. These differences reflect both ongoing uncertainty in the evidence base and variation in patient characteristics, clinical expertise, monitoring capabilities, and healthcare resources.

Although individual studies have examined specific aspects of mechanical ventilation in TBI, the evidence remains distributed across randomized trials, observational cohorts, and surveys conducted in diverse clinical settings. Differences in study populations, ventilation protocols, outcome measures, and follow-up periods make direct comparison challenging and have contributed to uncertainty regarding the optimal balance between lung-protective and brain-protective ventilation. An updated synthesis is therefore warranted to critically evaluate contemporary evidence, identify areas of agreement and conflicting findings, and clarify important gaps in current knowledge.

Accordingly, this systematic review was undertaken to evaluate contemporary evidence regarding mechanical ventilation practices in adult patients with TBI. The review focused on ventilatory strategies, lung-protective ventilation, airway management, tracheostomy practices, weaning approaches, ventilator-associated complications, and clinically relevant neurological and pulmonary outcomes. By synthesizing evidence across different study designs and healthcare settings, including evidence from LMICs, this review aims to provide a balanced assessment of current practice and identify priorities for future research.

## Review

Methods

Study Design

This systematic review was conducted and reported in accordance with the Preferred Reporting Items for Systematic Reviews and Meta-Analyses (PRISMA) 2020 statement to ensure methodological rigor, transparency, and reproducibility [10]. The review aimed to systematically evaluate contemporary evidence regarding mechanical ventilation practices in adult patients with TBI, with particular emphasis on ventilatory strategies, airway management, ICP optimization, duration of mechanical ventilation, weaning approaches, ventilator-associated complications, and clinically relevant patient outcomes [11,12].

Review Question

The review question was formulated using the Population, Intervention, Comparison, and Outcome (PICO) framework. The study population comprised adult patients with TBI requiring invasive mechanical ventilation. Interventions of interest included different ventilation strategies, ventilator settings, lung-protective ventilation, airway management techniques, tracheostomy, sedation practices, and weaning protocols. Comparator groups, where available, included standard care or alternative ventilatory approaches. The primary outcomes included mortality, neurological recovery, duration of mechanical ventilation, intensive care unit (ICU) length of stay, ventilator-associated complications, ICP, CPP, oxygenation indices, and other clinically relevant outcomes.

Literature Search

A comprehensive and systematic literature search was conducted across multiple electronic databases, including PubMed, Scopus, Web of Science, EBSCOhost, Cochrane Central Register of Controlled Trials (CENTRAL), Cumulative Index to Nursing and Allied Health Literature (CINAHL), EMBASE, and Google Scholar. Studies published from January 2000 to June 2026 were considered for eligibility.

The search strategy was designed to identify studies investigating mechanical ventilation practices and clinical outcomes in patients with TBI. Combinations of keywords, Medical Subject Headings (MeSH), and Boolean operators (“AND,” “OR”) were used to maximize search sensitivity and specificity. Search terms included “traumatic brain injury,” “TBI,” “head injury,” “severe traumatic brain injury,” “mechanical ventilation,” “ventilatory support,” “positive pressure ventilation,” “lung protective ventilation,” “airway management,” “endotracheal intubation,” “tracheostomy timing,” “weaning,” “ventilator-associated pneumonia,” “intracranial pressure,” “cerebral perfusion pressure,” “hyperventilation,” “hypoventilation,” “critical care,” “intensive care unit,” “lung protective,”“tidal volume,” “PEEP,” “CENTER-TBI,” “VENTIBRAIN” and “neurocritical care.” Synonymous terms were combined using the Boolean operator “OR,” while distinct concepts were linked using “AND” to ensure a comprehensive retrieval of relevant literature. Detailed search strategies for each database are provided in the supplementary material [12-14].

Eligibility Criteria

Studies were considered eligible if they enrolled adult patients with TBI receiving invasive mechanical ventilation or evaluated airway management strategies in TBI and reported outcomes related to ventilatory management or ventilation practices. Studies enrolling mixed acute brain injury populations were also considered eligible when patients with TBI were included and the reported ventilation-related findings were directly relevant to the review question. For such studies, findings were interpreted as mixed-population or contextual evidence rather than as evidence derived exclusively from TBI populations. Eligible study designs included randomized controlled trials (RCTs), prospective or retrospective cohort studies, case-control studies, and analytical cross-sectional studies published in peer-reviewed journals between January 2000 and June 2026. Only full-text articles published in English were included.

Studies were excluded if they were review articles, systematic reviews, meta-analyses, editorials, conference abstracts without complete manuscripts, case reports, expert opinions, study protocols, animal experiments, or laboratory-based investigations. Studies involving pediatric populations, non-traumatic neurological disorders, or those not reporting ventilation-related outcomes were also excluded. Duplicate publications and articles for which full-text versions could not be obtained despite reasonable efforts were excluded from the final analysis.

Study Selection

All records retrieved from the electronic databases and grey literature sources were imported into reference management software, where duplicate citations were identified and removed before screening. Two reviewers independently screened titles and abstracts against the predefined eligibility criteria. Potentially eligible articles subsequently underwent full-text assessment by the same reviewers. Disagreements regarding study eligibility were resolved through discussion, and when consensus could not be reached, a third reviewer was consulted. Reasons for exclusion during full-text review were documented to ensure transparency and reproducibility of the selection process.

Data Extraction

Data extraction was independently performed by two reviewers using a standardized data extraction form developed before study selection. Extracted variables included study characteristics (author, publication year, country, study design, sample size, participant characteristics, and severity of TBI) together with details of ventilatory management, including ventilation mode, tidal volume, PEEP, fraction of inspired oxygen (FiO₂), airway management strategies, tracheostomy timing, duration of mechanical ventilation, and weaning protocols. Clinical outcomes including mortality, neurological recovery, ICP, CPP, ventilator-associated complications, ICU length of stay, and hospital length of stay were extracted whenever reported. Discrepancies in extracted data were resolved through discussion until consensus was achieved.

Quality Assessment and Certainty of Evidence

The methodological quality and risk of bias of the included studies were independently assessed by two reviewers using validated appraisal tools appropriate to each study design. Observational studies (case-control, and cohort studies) were assessed using the Newcastle-Ottawa Scale (NOS), which examines the quality of participant selection, comparability of study groups, and ascertainment of exposure and outcomes. RCTs were assessed using the Cochrane Risk of Bias 2 (RoB 2) tool, which evaluates bias arising from the randomization process, deviations from intended interventions, missing outcome data, outcome measurement, and selection of the reported results. Quality assessment of included cross-sectional studies was done using the Joanna Briggs Institute (JBI) Critical Appraisal Checklist for Analytical Cross-Sectional Studies. All assessments were conducted independently by two reviewers. Any discrepancies in scoring or interpretation were resolved through discussion and consensus. When necessary, a third reviewer was consulted to reach a final decision. The results of the quality assessment were considered during data synthesis and interpretation to ensure a comprehensive evaluation of the quality of evidence regarding mechanical ventilation practices and clinical outcomes in patients with TBI. The certainty of evidence was assessed using the Grading of Recommendations Assessment, Development, and Evaluation (GRADE) framework, which evaluates domains including risk of bias, inconsistency, indirectness, imprecision, and publication bias. Based on these criteria, the quality of evidence was classified as high, moderate, low, or very low to ensure transparent interpretation of the findings [14-17].

Data Synthesis

Because of substantial clinical and methodological heterogeneity across the included studies with respect to study design, patient characteristics, ventilatory interventions, and reported outcomes, a quantitative meta-analysis was not considered appropriate. Therefore, findings were synthesized narratively. The evidence was organized into predefined thematic categories, including ventilatory strategies, airway management, lung-protective ventilation, ICP optimization, tracheostomy practices, ventilator-associated complications, weaning strategies, and clinical outcomes. Areas of agreement, inconsistencies, and remaining evidence gaps were critically examined to provide an integrated interpretation of the available evidence.

Ethical Considerations and Registration

As this review synthesized data exclusively from previously published studies, institutional ethical approval and informed consent were not required. The review protocol was prospectively registered with the International Prospective Register of Systematic Reviews (PROSPERO) under registration number CRD420261456801.

Results

Study Selection

The search strategy identified 1,864 records from electronic databases and 48 records from other sources, including grey literature and trial registries. Before screening, 1,021 duplicate records and 142 records removed for other reasons were excluded, leaving 701 records for title and abstract screening. Following title and abstract screening, 569 records were excluded, and 132 reports were sought for retrieval. Of these, 95 reports could not be retrieved, leaving 37 full-text reports for eligibility assessment. Of the 37 reports assessed, 26 were excluded because of an inappropriate study population (non-TBI population, n=12), inappropriate intervention or exposure (mechanical ventilation or ventilation strategies not evaluated, n=8), or failure to report predefined ventilation-related outcomes (n=6). An additional 48 records were identified through other sources. Of these, 36 reports could not be retrieved, while 12 reports underwent full-text eligibility assessment. All 12 reports were excluded because they did not report mechanical ventilation-related outcomes (n=7), reported outcomes that were not aligned with the objectives of the review (n=3), or evaluated interventions unrelated to mechanical ventilation practices in patients with TBI (n=2). Ultimately, 11 studies, corresponding to 11 reports, met the predefined eligibility criteria and were included in the qualitative synthesis [18-28] (Figure 1).

**Figure 1:**
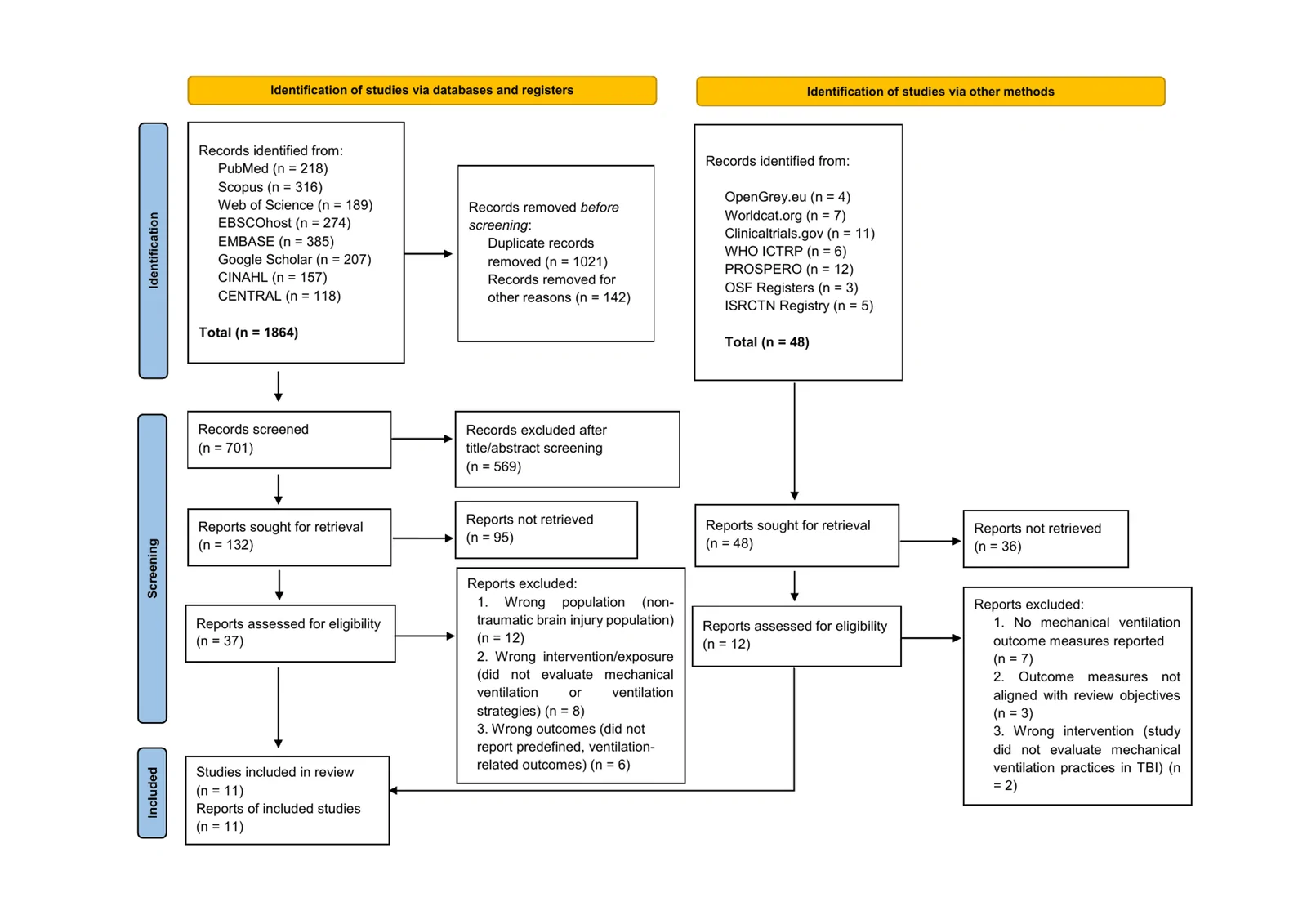
PRISMA 2020 flow diagram illustrating the study selection process for the systematic review PRISMA 2020 flow diagram depicting the study selection process. The literature search identified records through electronic databases and additional sources, followed by duplicate removal, title and abstract screening, full-text eligibility assessment, and final inclusion of studies in the qualitative synthesis. Reasons for exclusion at the full-text screening stage are summarized within the diagram. The review was conducted in accordance with the Preferred Reporting Items for Systematic Reviews and Meta-Analyses (PRISMA) 2020 guidelines.

Characteristics of Included Studies

The included studies demonstrated substantial heterogeneity in study design, geographical setting, sample size, and primary focus of mechanical ventilation and airway management in patients with TBI. The studies were conducted across India, Brazil, China, Canada, Italy, Europe, and multinational settings, with sample sizes ranging from 53 to 13,925 participants. Both observational and interventional designs were represented, including prospective and retrospective cohort studies, multicentre observational studies, cross-sectional surveys, and RCTs. The primary areas of investigation included tracheostomy, prehospital and in-hospital intubation, ventilator management practices, partial pressure of carbon dioxide in arterial blood (PaCO₂) and hyperventilation, lung-protective ventilation, ventilation parameters, and extubation failure. Survey-based studies consistently demonstrated considerable variability and heterogeneity in ventilation practices and ventilator targets across ICUs, particularly in LMICs. Interventional studies reported mixed findings, with lung-protective ventilation showing potential benefits for oxygenation and pulmonary outcomes in some settings, while fixed lung-protective ventilation was associated with worse composite outcomes in another trial. Observational studies further indicated that airway-management strategies, including early tracheostomy and appropriate timing of intubation, may influence neurological and clinical outcomes. Overall, the included evidence reflects considerable variation in mechanical ventilation and airway-management practices in TBI, with emerging evidence supporting individualized ventilation strategies while highlighting the need for standardized, evidence-based protocols (Table 1).

**Table 1:** Characteristics and key findings of the studies included in the systematic review The table summarizes the characteristics of the 11 studies included in the qualitative synthesis. These include the country, study design, study population, ventilation-related intervention or focus, and the principal findings regarding mechanical ventilation practices in adult patients with traumatic brain injury (TBI). ABI, acute brain injury; GCS, Glasgow Coma Scale; ICP, intracranial pressure; ICU, intensive care unit; LMICs, low- and middle-income countries; PaCO₂, arterial partial pressure of carbon dioxide; PEEP, positive end-expiratory pressure; RCT, randomized controlled trial.

Author (Year)	Country	Study Design	Sample Size	Primary Focus	Key Findings
Sundaram et al., 2018 [18]	India	Prospective observational	53	Early tracheostomy without routine mechanical ventilation	Early tracheostomy with best medical management achieved outcomes comparable to published ICU cohorts despite the absence of routine mechanical ventilation and ICP monitoring.
Gravesteijn et al., 2020 [19]	Europe	Multicentre prospective observational cohort (CENTER-TBI)	4,509	Prehospital vs. inhospital intubation	In-hospital intubation benefited patients with severe neurological injury (GCS ≤10).
Picetti et al., 2020 [20]	International	Cross-sectional survey	687	Ventilation Practices	Considerable variability in ventilator settings and PaCO₂targets across ICUs.
Jiang et al., 2021 [21]	China	Randomized controlled trial	90	Lung-protective Ventilation	Improved oxygenation and pulmonary outcomes without increasing ICP.
Mascia et al., 2024 [22]	Italy	Multicentre randomized clinical trial	190	Lung-protective Ventilation	Fixed lung-protective ventilation was associated with worse composite outcomes than conventional ventilation.
Prabhakar et al., 2025 [23]	Multinational (LMICs)	Cross-sectional survey	204	Ventilator Management Practices	Marked heterogeneity in ventilation protocols across LMICs
Robba et al., 2025 [24]	Multinational	Prospective Cohort	2,095	Ventilation Parameters	Lung-protective ventilation was commonly adopted butpractices varied considerably.
Lu et al., 2026 [25]	Canada	Retrospective registry-based cohort	13,925	Ventilation practices in ABI	Lower PEEP was commonly used in ABI patients despite similar tidal volumes
Citerio et al., 2021 [26]	Multicentre; Europe/International (CENTER-TBI)	Secondary analysis of a prospective, multicentre, observational cohort study	1,100	Use of hyperventilation and its relationship with intracranial pressure (ICP) and outcomes	Management in centres using profound hyperventilation (PaCO₂ <30 mmHg) more frequently was not associated with increased 6-month mortality or unfavourable neurological outcome.
Robba et al., 2020 [27]	Multicentre; Europe/International (CENTER-TBI)	Prospective, multicentre, observational longitudinal cohort study	1,358	Tracheostomy practice and timing in TBI	The authors concluded that early tracheostomy was associated with better neurological outcome and reduced length of stay, although causality could not be established.
Reis et al., 2013 [28]	Brazil	Prospective cohort study	311	Extubation failure in TBI and its association with clinical and functional outcomes	Extubation failure was associated with higher in-hospital mortality (20.9% vs. 4.5%), greater need for tracheostomy (55.8% vs. 1.9%), and longer hospital stay (44 vs. 27 days)

Among the 11 included studies, most focused specifically on patients with TBI, whereas three studies enrolled broader acute brain injury populations that included patients with TBI. These mixed-population studies were retained because their ventilation-related findings were directly relevant to the review question and were interpreted cautiously as contextual evidence rather than as TBI-specific evidence.

Quality Assessment

Overall, the methodological quality of the included studies ranged from moderate to high. Most RCTs demonstrated a low risk of bias across key methodological domains, whereas observationalstudies achieved satisfactory NOS scores. Common methodological limitations includedrelatively small sample sizes, variability in ventilatory protocols, heterogeneity in outcome definitions, andinconsistent reporting of neurological outcomes. Despite these limitations, the overall quality of evidence was considered adequate to permit qualitative synthesis.

Table 2 presents the methodological quality assessment of the included observational studies using the NOS.

**Table 2:** Newcastle-Ottawa Scale (NOS) quality assessment of included cohort studies

Study (Year)	Study design	Selection (4)	Comparability (2)	Outcome/Follow-up (3)	NOS score	Overall quality
Sundaram et al., 2018 [18]	Prospective observational cohort	3	0	2	5/9	Moderate
Gravesteijn et al., 2020 [19] (CENTER-TBI)	Multicenter prospective cohort	4	2	3	9/9	High
Robba et al., 2025 [24] (VENTIBRAIN)	Multicenter prospective cohort	4	2	3	9/9	High
Lu et al., 2026 [25]	Retrospective registry cohort	4	2	2	8/9	High
Citerio et al., 2021 [26]	prospective multicentre observational cohort	3	2	3	8/9	High
Robba et al., 2020 [27]	Prospective multicentre observational cohort	3	2	3	8/9	High
Reis et al., 2013 [28]	Prospective cohort study	4	2	3	9/9	High

The studies were assessed across three domains: Selection (maximum four points), Comparability (maximum two points), and Outcome/Follow-up (maximum three points), giving a maximum possible score of nine points. NOS scores ranged from five to nine out of nine, with studies classified as moderate or high quality. Most studies demonstrated high methodological quality, scoring eight or nine out of nine, while Sundaram et al. (2018) had a comparatively lower score of five out of nine, indicating moderate methodological quality. Overall, the included evidence was predominantly of high methodological quality, with adequate participant selection, comparability, and outcome assessment/follow-up.

Two RCTs were evaluated using the Cochrane RoB 2 tool as shown in Table 3.

**Table 3:** Cochrane Risk of Bias 2 (RoB 2) assessment of the included randomized controlled trials

Study	Randomization process	Deviations from Intended Interventions	Missing outcome data	Outcome measurement	Selection of reported results	Overall risk of bias
Jiang et al., 2021 [21]	Low	Low	Low	Low	Some concerns	Low
Mascia et al., 2024 [22]	Low	Low	Low	Low	Low	Low

Overall, the included trials demonstrated a low risk of bias across most methodological domains. Jiang et al., 2021 [21] and Mascia et al., 2024 [22] were judged to have an overall low risk of bias. No trial was considered to have a high risk of bias in any domain.

Two cross-sectional studies were evaluated using the JBI critical appraisal checklist as seen in Table 4.

**Table 4:** Joanna Briggs Institute (JBI) critical appraisal of the included cross-sectional studies

Study	Study design	JBI score (8)	Overall quality	Key limitation
Picetti et al., 2020 [20]	International cross-sectional survey	7/8	High	Confounding factors not explicitly identified before analysis.
Prabhakar et al., 2025 [23]	International cross-sectional survey	8/8	High	No significant methodological limitations.

Both studies demonstrated high methodological quality. Prabhakar et al., 2025 [23] achieved the maximum score of eight out of eight, fulfilling all appraisal criteria, whereas Picetti et al., 2020 [20] scored seven out of eight because potential confounding factors were not explicitly identified before statistical analysis. Overall, both studies were considered to have a low risk of methodological bias and were included in the qualitative synthesis.

Certainty of Evidence

Table 5 presents the GRADE assessment of the certainty of evidence for the included studies evaluating mechanical ventilation and airway-management strategies in patients with TBI.

**Table 5:** Certainty of evidence assessment using GRADE GRADE: Grading of Recommendations Assessment, Development, and Evaluation.

Author (Year)	Study design	Risk of bias	Inconsistency	Indirectness	Imprecision	Publication bias	Overall GRADE certainty
Sundaram et al., 2018 [18]	Prospective observational study	Serious	Not serious	Serious	Serious	Undetected	Low
Gravesteijn et al., 2020 [19]	Multicenter prospective observational cohort (CENTER-TBI)	Serious	Not serious	Not serious	Not serious	Undetected	Moderate
Picetti et al., 2020 [20]	International cross-sectional survey	Serious	Serious	Serious	Not serious	Likely	Very Low
Jiang et al., 2021 [21]	Randomized Controlled Trial	Some concerns	Not serious	Not serious	Serious	Undetected	Moderate
Mascia et al., 2024 [22]	Multicenter Randomized Controlled Trial	Some concerns	Not serious	Not serious	Serious	Undetected	Moderate
Prabhakar et al., 2025 [23]	International cross-sectional survey	Serious	Serious	Serious	Not serious	Likely	Very Low
Robba et al., 2025 [24]	Multicenter prospective observational study	Serious	Not serious	Not serious	Not serious	Undetected	Moderate
Lu et al., 2026 [25]	Registry-based retrospective cohort study	Serious	Not serious	Not serious	Not serious	Undetected	Low
Citerio et al., 2021 [26]	prospective multicentre observational cohort	Moderate	Low	Low	Moderate	Low	Moderate
Robba et al., 2020 [27]	Prospective multicentre observational cohort	Moderate	Low	Low	Moderate	Low	Moderate
Reis et al., 2013 [28]	Prospective cohort study	Moderate	Low	Low	Moderate	Low	Moderate

The evidence was assessed across five domains: risk of bias, inconsistency, indirectness, imprecision, and publication bias. Overall certainty ranged from very low to moderate. The cross-sectional survey studies by Picetti et al., 2020 [20] and Prabhakar et al., 2025 [23] demonstrated very low certainty, primarily due to serious risk of bias, inconsistency, indirectness, and likely publication bias. Sundaram et al., 2018 [18] was graded as low certainty, while Gravesteijn et al., 2020 [19], Jiang et al., 2021 [21], Mascia et al., 2024 [22], Robba et al., 2025 [24], Citerio et al., 2021 [26] , Robba et al., 2020 [27], and Reis et al., 2013 [28] were assessed as having moderate certainty, with limitations mainly related to risk of bias and/or imprecision. Overall, the findings suggest that although several studies provide reasonably reliable evidence, confidence in the evidence is limited by methodological and precision-related concerns, supporting the need for further high-quality prospective and randomized studies.

Result Summary

The included studies showed substantial heterogeneity in design, setting, sample size, and mechanical ventilation/airway-management strategies in TBI. Key areas included tracheostomy, intubation timing, ventilator management, PaCO₂/hyperventilation, lung-protective ventilation, and extubation failure. Overall, the findings support individualized ventilation strategies, while highlighting the need for standardized protocols. Methodological quality was generally moderate to high. Observational studies had NOS scores of five to nine out of nine, RCTs showed low overall risk of bias, and cross-sectional studies demonstrated high quality on JBI appraisal. The GRADE certainty of evidence ranged from very low to moderate. Most evidence was rated moderate, whereas the cross-sectional studies had very-low certainty because of concerns related to bias, inconsistency, indirectness, and publication bias. Overall, further high-quality prospective and randomized studies are needed.

Synthesis of Findings

Considerable variation in mechanical ventilation practices was observed across the included studies. Lung-protective ventilation employing lower tidal volumes was the most frequently reported strategy, while individualized optimization of PEEP was commonly recommended to maintain adequate oxygenation without exacerbating secondary brain injury. Studies also highlighted substantial variability in airway management strategies, timing of tracheostomy, and weaning protocols across different institutions and healthcare systems. Several studies suggested that early tracheostomy was associated with shorter durations of mechanical ventilation and reduced ICU length of stay; however, evidence regarding improvements in mortality and long-term neurological outcomes remained inconsistent. Across the included literature, maintaining adequate oxygenation while avoiding both hypoxemia and excessive hyperventilation was consistently emphasized to optimize cerebral perfusion and minimize intracranial hypertension. Ventilator-associated pneumonia emerged as one of the most frequently reported complications among mechanically ventilated patients with TBI.

Overall, the available evidence supports an individualized approach to mechanical ventilation that integrates lung-protective strategies with established neurocritical care principles. Nevertheless, the considerable heterogeneity among studies and the limited availability of high-quality Indian data highlight the need for well-designed multicenter prospective studies to establish standardized, evidence-based ventilation protocols for patients with TBI.

Discussion

Mechanical ventilation is an integral component of neurocritical care in patients with moderate-to-severe TBI, with the dual objectives of maintaining adequate gas exchange and minimizing secondary cerebral injury. The present systematic review synthesized evidence from 11 eligible studies encompassing RCTs, observational cohort studies, and international cross-sectional surveys. Overall, the evidence demonstrated substantial variation in ventilatory strategies, airway management, tracheostomy practices, ventilator targets, and neuromonitoring approaches. Despite this heterogeneity, the findings broadly support an individualized approach in which pulmonary protection is balanced against cerebral physiological considerations rather than applying a uniform ventilation protocol to all patients with TBI. This interpretation is consistent with contemporary neurocritical care principles emphasizing maintenance of adequate oxygenation and cerebral perfusion while avoiding potentially harmful extremes of carbon dioxide and airway pressures [3,4].

A principal finding of this review was the increasing use of lung-protective ventilation in patients with TBI and acute brain injury. Historically, clinicians have been cautious about applying lower tidal volumes and higher PEEP in patients with intracranial pathology because increases in intrathoracic pressure may influence cerebral venous drainage, ICP, and CPP. However, the available contemporary evidence suggests that pulmonary-protective strategies can be considered when accompanied by appropriate assessment of cerebral physiology. Jiang et al. reported improved pulmonary compliance and oxygenation and fewer pulmonary complications with a strategy incorporating lower tidal volumes and moderate PEEP, without a clinically important adverse effect on ICP [21]. These findings suggest that pulmonary and cerebral protection are not necessarily mutually exclusive goals, provided that ventilatory adjustments are accompanied by appropriate neurological and respiratory monitoring.

Importantly, the randomized trials did not produce entirely consistent findings. While Jiang et al. [21] reported favorable pulmonary effects with a lower tidal volume and PEEP-based strategy, the multicenter Lung-Protective Mechanical Ventilation in Patients with Severe Acute Brain Injury (PROLABI) trial by Mascia et al. [22] reported less favorable outcomes with a fixed lung-protective ventilation protocol, including a higher composite adverse outcome and mortality. These apparently contrasting findings are clinically important and argue against assuming that conventional lung-protective targets are universally appropriate in patients with severe TBI. Differences in patient characteristics, ventilation protocols, PEEP levels, carbon dioxide management, and the degree of cerebral monitoring may partly explain the divergent findings. The findings therefore support a physiology-guided approach in which tidal volume and PEEP are adjusted according to respiratory mechanics, gas exchange, ICP/CPP where available, and the patient's overall neurological status rather than applied as rigid targets.

The observational evidence further illustrates the complexity of ventilatory management in TBI. Data from the Collaborative European NeuroTrauma Effectiveness Research in Traumatic Brain Injury (CENTER-TBI) cohort indicated that prehospital intubation was not associated with a universal improvement in functional outcome after adjustment for relevant factors, although selected patients with severe neurological impairment appeared to derive greater benefit from appropriate airway management [19]. Similarly, the Multicentre Observational Study on Practice of Ventilation in Brain Injured Patients (VENTIBRAIN) study demonstrated widespread use of lung-protective ventilation principles but considerable international variation in tidal volume, PEEP, oxygenation targets, and other ventilator parameters [24]. These findings should be interpreted cautiously because observational studies cannot reliably establish whether differences in ventilation caused differences in outcomes. Nevertheless, they provide important information about real-world practice and demonstrate that clinician judgment, injury severity, institutional protocols, and available resources continue to influence ventilatory management.

The international surveys by Picetti et al. and Prabhakar et al. further demonstrated substantial variability in contemporary practice [20,23]. Differences were observed in ventilator settings, arterial carbon dioxide targets, recruitment maneuvers, PEEP selection, and the availability of advanced neuromonitoring. Such variation was particularly relevant across LMICs, where differences in critical care infrastructure, staffing, monitoring capacity, and institutional resources may influence clinical decision-making. These findings highlight a potential gap between general guideline recommendations and their implementation in diverse healthcare environments. Rather than advocating a single rigid protocol, future recommendations should provide evidence-based physiological targets while allowing adaptation to patient characteristics and resource availability.

Carbon dioxide management represents another important area in which pulmonary and cerebral considerations must be balanced. Reduction of arterial carbon dioxide can decrease cerebral blood flow through cerebral vasoconstriction, whereas excessive carbon dioxide may increase cerebral blood volume and potentially worsen intracranial hypertension. Consequently, prolonged or indiscriminate hyperventilation should generally be avoided, while ventilation should be adjusted according to the patient's neurological condition and ICP status when monitoring is available [5,6]. The variation in carbon dioxide targets identified in the included surveys reinforces the need for further research to determine whether individualized carbon dioxide targets based on cerebral physiology can improve patient-centered outcomes.

Airway management is similarly important because both inadequate ventilation and complications associated with airway intervention can contribute to secondary brain injury. Early endotracheal intubation may be appropriate in patients with impaired consciousness, loss of airway protective reflexes, severe hypoxemia, or inadequate ventilation. However, the available evidence does not support indiscriminate intubation solely on the basis of TBI diagnosis or injury severity. The CENTER-TBI findings suggest that the potential benefit of airway intervention may differ according to neurological severity and clinical context [19]. During airway management, particular attention should therefore be given to avoiding peri-intubation hypoxemia and hypotension, which may further compromise cerebral perfusion.

The timing of tracheostomy remains another area of uncertainty. The study by Sundaram et al. provided evidence regarding early tracheostomy in a resource-limited setting, whereas broader evidence suggests that early tracheostomy may facilitate airway care and reduce the duration of mechanical ventilation or ICU stay in selected patients [18,29]. However, the evidence does not consistently demonstrate reductions in mortality or improvement in long-term neurological recovery. Tracheostomy should therefore be considered on an individual basis, taking into account the anticipated duration of ventilatory support, neurological prognosis, airway protection, secretion management, sedation requirements, and the resources available for post-acute care.

Ventilator-associated pulmonary complications remain an important concern in mechanically ventilated patients with TBI. Pulmonary complications may prolong mechanical ventilation and ICU stay and can interfere with neurological rehabilitation. The included studies support attention to lung-protective ventilation, appropriate sedation practices, meticulous airway care, early mobilization when clinically feasible, and established infection-prevention measures [21,22,24]. Importantly, these measures should be implemented without compromising cerebral perfusion or neurological monitoring. A multidisciplinary approach involving intensivists, neurosurgeons, respiratory therapists, physiotherapists, and nursing personnel is therefore particularly important in this population. 

Recent evidence further supports the importance of individualized ventilatory and airway management in TBI. Citerio et al. [26] highlighted the clinical relevance of careful PaCO₂ management and avoidance of inappropriate hyperventilation in mechanically ventilated patients with TBI. Robba et al. [27] provided additional TBI-specific evidence regarding tracheostomy practice and timing, supporting the need to consider tracheostomy within the context of neurological prognosis and anticipated ventilator dependence. Furthermore, Reis et al. [28] reported that extubation failure was associated with higher mortality, increased need for tracheostomy, and prolonged hospital stay, emphasizing the importance of careful assessment of readiness for extubation in patients with TBI.

The findings of this review also have relevance for resource-constrained healthcare systems. Although several contemporary studies were conducted in high-resource settings, relatively limited evidence was available from India and other LMICs. The international survey data indicate that access to ICP monitoring, advanced neuromonitoring, specialized neurocritical care personnel, and standardized protocols varies considerably between institutions [23]. Consequently, ventilation recommendations developed predominantly in high-resource environments may not be directly transferable to all settings. Future research from India and other LMICs should therefore evaluate feasible ventilation strategies within the context of locally available monitoring and critical care resources.

Taken together, the evidence does not identify a single ventilation strategy that can be considered optimal for all patients with TBI. Instead, the findings support an individualized model in which ventilatory management is continuously adjusted according to oxygenation, carbon dioxide levels, respiratory mechanics, hemodynamics, ICP/CPP when available, and neurological status. The conflicting results of the available randomized trials are particularly important because they demonstrate that the theoretical benefits of lung protection cannot be separated from their potential cerebral consequences. Thus, the principal clinical question is not simply whether lung-protective ventilation should be used, but how and in whom it should be applied while preserving cerebral physiology.

*Practical Considerations* *for Clinical Practice*

Based on the available evidence, several practical considerations can be proposed. First, mechanical ventilation should be individualized according to the severity of brain injury, respiratory mechanics, gas exchange, hemodynamic status, and available neuromonitoring rather than applied through a single fixed protocol. Second, lower tidal volume ventilation may be considered when clinically appropriate, but its implementation should be accompanied by monitoring of arterial carbon dioxide, oxygenation, and cerebral physiology, particularly in patients at risk of intracranial hypertension. Third, PEEP should be titrated according to oxygenation and respiratory mechanics while considering its potential effects on intrathoracic pressure, venous return, ICP, and CPP. Fourth, prolonged prophylactic hyperventilation should be avoided, while temporary hyperventilation may be considered in appropriately selected patients with acute intracranial hypertension as part of broader emergency management [5,6]. Fifth, airway interventions should prioritize prevention of hypoxemia and hypotension, with intubation decisions based on clinical indications rather than TBI diagnosis alone. Finally, tracheostomy and weaning decisions should be individualized according to neurological prognosis, expected duration of ventilatory support, secretion burden, airway protection, and institutional resources. In resource-limited settings, practical implementation should emphasize fundamental principles such as prevention of hypoxemia and hypotension, appropriate tidal volume selection, careful PEEP adjustment, avoidance of unnecessary hyperventilation, standardized airway-care practices, and systematic monitoring of available physiological parameters. These measures may provide a pragmatic framework while more advanced neuromonitoring facilities are unavailable.

Limitations

This systematic review has several limitations. First, only 11 studies met the predefined eligibility criteria, limiting the volume and strength of available evidence. Second, substantial clinical and methodological heterogeneity existed in study design, patient characteristics, severity of brain injury, ventilatory interventions, monitoring practices, outcome definitions, and follow-up, precluding quantitative meta-analysis and limiting direct comparisons. Third, much of the evidence came from observational cohorts and cross-sectional surveys, which are susceptible to selection bias, residual confounding, and reporting bias and cannot establish causality. Fourth, definitions and implementation of lung-protective ventilation, PEEP strategies, airway interventions, and neurological outcomes varied considerably, potentially contributing to conflicting findings. Fifth, some studies, including Mascia et al., Robba et al. (2025), and Lu et al., enrolled mixed acute brain injury populations rather than TBI exclusively. These findings were therefore interpreted cautiously, particularly where TBI-specific subgroup data were limited. Sixth, the limited representation of India and other LMICs may restrict generalizability to resource-constrained settings. Seventh, restriction to English-language publications may have introduced language and publication bias. Eighth, some potentially relevant reports could not be retrieved despite reasonable efforts, creating a potential risk of retrieval bias. Finally, evolving mechanical ventilation practices and neurocritical care approaches mean that earlier findings may not fully reflect contemporary practice. Overall, these limitations warrant cautious interpretation and indicate that the findings should be viewed as a synthesis of available evidence rather than definitive evidence of superiority of any specific ventilatory strategy.

Future Directions

Future research should prioritize adequately powered, multicenter RCTs designed specifically to evaluate the interaction between pulmonary and cerebral physiology during mechanical ventilation in TBI. Particular attention should be directed toward individualized PEEP titration, optimal tidal volume selection, arterial carbon dioxide targets, oxygenation targets, and the role of recruitment maneuvers in patients with varying degrees of intracranial compliance and pulmonary dysfunction. Future studies should also incorporate standardized neurological and patient-centered outcomes, including long-term functional recovery rather than relying predominantly on mortality or short-term ICU outcomes. Integration of ICP, CPP, cerebral oxygenation, respiratory mechanics, and advanced neuromonitoring may help identify which patients are most likely to benefit from particular ventilatory strategies. Comparative research should also evaluate different approaches to tracheostomy timing, ventilator weaning, sedation, and airway management. Finally, high-quality prospective studies from India and other LMICs are particularly important. Such studies should evaluate ventilation strategies within real-world resource constraints and assess whether simplified, standardized care pathways can improve outcomes where advanced neuromonitoring is not universally available. Collaborative multicenter research could ultimately help establish flexible, physiology-guided ventilation recommendations that are both evidence-based and applicable across different healthcare environments.

## Conclusions

Mechanical ventilation is a critical component of neurocritical care in patients with TBI, requiring a careful balance between adequate pulmonary support and preservation of cerebral physiology. The findings of this systematic review suggest that contemporary ventilatory management should be individualized, with lung-protective principles applied cautiously while maintaining adequate oxygenation, avoiding prolonged or unnecessary hyperventilation, and considering ICP and cerebral perfusion. Although early tracheostomy and appropriate airway management may reduce the duration of mechanical ventilation and ICU stay in selected patients, the available evidence remains heterogeneous and does not establish consistent benefits in mortality or long-term neurological recovery. The limited availability of high-quality evidence from India and other resource-constrained settings represents an important gap in the current literature. Further well-designed multicenter RCTs and prospective cohort studies are needed to define optimal ventilatory targets, evaluate physiology-guided ventilation strategies, and determine their effects on long-term neurological and pulmonary outcomes. Overall, the available evidence supports standardized physiological safety principles with patient-specific adjustment of ventilatory settings, integrating pulmonary and cerebral physiological considerations to minimize secondary brain injury and optimize patient outcomes.

## Appendices

**Table 6:** Literature search strategy Mesh, Medical Subject Headings; MH, Major Heading; TI, Title; AB, Abstract; TS, Topic Search; TBI, traumatic brain injury; OSF, Open Science Framework; WHO ICTRP, World Health Organization International Clinical Trials Registry Platform.

Database	Search Strategy	Filters Applied
PubMed	("Brain Injuries, Traumatic"[Mesh] OR "Craniocerebral Trauma"[Mesh] OR "traumatic brain injury"[tiab] OR TBI[tiab] OR "head injury"[tiab] OR "brain injury"[tiab] OR "severe traumatic brain injury"[tiab] OR "craniocerebral trauma"[tiab] OR "acute brain injury"[tiab] OR neurotrauma[tiab]) AND ("Respiration, Artificial"[Mesh] OR "Ventilators, Mechanical"[Mesh] OR "mechanical ventilation"[tiab] OR "invasive mechanical ventilation"[tiab] OR "artificial ventilation"[tiab] OR "pulmonary ventilation"[tiab] OR ventilator[tiab] OR "ventilatory support"[tiab] OR "respiratory support"[tiab] OR "lung protective ventilation"[tiab] OR "positive pressure ventilation"[tiab] OR "ventilator management"[tiab] OR extubation[tiab] OR weaning[tiab])	English; Humans; Publication period: January, 2000 to June, 2026
Scopus	TITLE-ABS-KEY (("traumatic brain injury" OR TBI OR "head injury" OR "brain injury" OR "craniocerebral trauma" OR "acute brain injury" OR neurotrauma) AND ("mechanical ventilation" OR "invasive mechanical ventilation" OR "artificial ventilation" OR "pulmonary ventilation" OR ventilator OR "ventilatory support" OR "respiratory support" OR "lung protective ventilation" OR "positive pressure ventilation" OR "ventilator management" OR extubation OR weaning))	English; Articles, conference papers, and clinical studies; 2000 to search date
Web of Science	TS=(("traumatic brain injury" OR TBI OR "head injury" OR "brain injury" OR "craniocerebral trauma" OR "acute brain injury") AND ("mechanical ventilation" OR "invasive mechanical ventilation" OR "artificial ventilation" OR ventilator OR "ventilatory support" OR "respiratory support" OR "lung protective ventilation" OR "positive pressure ventilation" OR extubation OR weaning))	English; 2000 to search date
EMBASE	('traumatic brain injury'/exp OR 'craniocerebral trauma'/exp OR 'traumatic brain injury' OR TBI) AND ('mechanical ventilation'/exp OR 'artificial ventilation'/exp OR 'mechanical ventilation' OR ventilator OR 'ventilatory support' OR 'positive pressure ventilation' OR 'lung protective ventilation')	English; 2000 to search date
EBSCOhost (MEDLINE/CINAHL)	(MH "Brain Injuries, Traumatic+" OR TI ("traumatic brain injury" OR TBI OR "head injury") OR AB ("traumatic brain injury" OR TBI OR "head injury")) AND (MH "Respiration, Artificial+" OR MH "Mechanical Ventilation+" OR TI ("mechanical ventilation" OR ventilator OR "artificial ventilation") OR AB ("mechanical ventilation" OR ventilator OR "artificial ventilation"))	English; Human studies
Cochrane CENTRAL	("traumatic brain injury" OR TBI OR "head injury" OR "brain injury") AND ("mechanical ventilation" OR "artificial ventilation" OR ventilator OR "ventilatory support" OR "positive pressure ventilation")	No additional filters
CINAHL	(MH "Brain Injuries, Traumatic+") AND (MH "Respiration, Artificial+" OR MH "Mechanical Ventilation+") OR ("traumatic brain injury" AND "mechanical ventilation")	English; Human studies
Google Scholar	"traumatic brain injury" AND "mechanical ventilation"; "TBI" AND ventilator; "head injury" AND "mechanical ventilation"; "severe traumatic brain injury" AND ventilation; "brain injury" AND "ventilator management"	First 200 most relevant records screened
Grey Literature	OpenGrey, WorldCat, ClinicalTrials.gov, WHO ICTRP, PROSPERO, Open Science Framework (OSF) Registries, and ISRCTN Registry were searched using combinations of the terms "traumatic brain injury", "TBI", "head injury", "mechanical ventilation", "ventilator", and "ventilatory support".	No language restriction where applicable; records screened manually
